# Impact of Edible Cricket Consumption on Gut Microbiota in Healthy Adults, a Double-blind, Randomized Crossover Trial

**DOI:** 10.1038/s41598-018-29032-2

**Published:** 2018-07-17

**Authors:** Valerie J. Stull, Elijah Finer, Rachel S. Bergmans, Hallie P. Febvre, Colin Longhurst, Daniel K. Manter, Jonathan A. Patz, Tiffany L. Weir

**Affiliations:** 1 Center for Sustainability and the Global Environment, University of Wisconsin-Madison, Madison, Wisconsin, USA; 2 Department of Food Science and Human Nutrition, Colorado State University, Fort Collins, CO USA; 3 Department of Psychiatry, School of Medicine, University of Michigan, Ann Arbor, Michigan, USA; 4 Department of Biostatistics and Medical Informatics, University of Wisconsin-Madison, Madison, Wisconsin, USA; 5 USDA-ARS-Soil Management and Sugarbeet Research, Fort Collins, CO 80523 USA

## Abstract

Edible insects are often considered a nutritious, protein-rich, environmentally sustainable alternative to traditional livestock with growing popularity among North American consumers. While the nutrient composition of several insects is characterized, all potential health impacts have not been evaluated. In addition to high protein levels, crickets contain chitin and other fibers that may influence gut health. In this study, we evaluated the effects of consuming 25 grams/day whole cricket powder on gut microbiota composition, while assessing safety and tolerability. Twenty healthy adults participated in this six-week, double-blind, crossover dietary intervention. Participants were randomized into two study arms and consumed either cricket-containing or control breakfast foods for 14 days, followed by a washout period and assignment to the opposite treatment. Blood and stool samples were collected at baseline and after each treatment period to assess liver function and microbiota changes. Results demonstrate cricket consumption is tolerable and non-toxic at the studied dose. Cricket powder supported growth of the probiotic bacterium, *Bifidobacterium animalis*, which increased 5.7-fold. Cricket consumption was also associated with reduced plasma TNF-α. These data suggest that eating crickets may improve gut health and reduce systemic inflammation; however, more research is needed to understand these effects and underlying mechanisms.

## Introduction

The human gastrointestinal tract is home to a host of bacterial cells. These cells outnumber human cells by a factor of three^1^ and encode at least 100 times more genes^2^, which influence human physiology, metabolism, and gene expression pertinent to immune function, energy, and even mood^3^. Extensive research demonstrates that microbiota in the gut respond to nutritional cues and generate hormone-like signals influencing normal physiology, nutritional status, metabolism, immune function, as well as disease progression and overall wellbeing^2,4–6^. Imbalances in the gut microbiota, also known as dysbiosis, and low microbial diversity are associated with metabolic and non-communicable diseases, gastrointestinal conditions, allergies, asthma, and even neuropsychiatric disorders^7–10^.

Diet is an especially relevant factor in defining the composition of gut microbiota^11^, and even small shifts have demonstrated meaningful effects^5,12^. Dietary diversity is linked with a more diverse, healthy microbiota that is more adept at adjusting to perturbations^13^. Indigestible dietary carbohydrates (dietary fibers) are the primary energy sources for gut microbiota, and thus shape microbial growth^14^. Not surprisingly, dietary fiber intake has been shown to contribute to the health of the gut microbiome by increasing diversity in fecal microbiota^15,16^, and high fiber intake has been associated with a reduced risk of breast cancer^17^, diverticular disease^18^, coronary heart disease^19,20^, and metabolic syndrome^21,22^. Edible insects are hailed as an excellent source of protein and other nutrients, but they also provide a relatively understudied fiber source, chitin, that could influence the gut microbiota. For western consumers, edible insects are a novel food that is just now gaining traction in certain areas.

Motivations to eat insects stem from their cultural and nutritional value, as well as their numerous environmental benefits. The current pressures on global food security, including climate change, population growth, and shifting dietary preferences, have ignited a search for more environmentally sustainable protein sources. Given that livestock production alone is responsible for about 14.5% of total human-induced greenhouse gas (GHG) emissions^23^ there is a mounting need for more efficient animal production systems. Edible insects have been touted as one such option, as they typically emit fewer GHGs^24^ and require less land, water, and feed to survive and thrive than traditional livestock^25^. The result is a significantly lower environmental impact^24–26^, and high desirability due in part to insects’ large edible body mass percentage^27^, high feed-conversion ratio^26^, and ectothermic thermoregulation, which limits energy expenditure on temperature regulation.

Entomophagy, the practice of eating insects, is not new however; it has been recorded throughout human history across the globe^28,29^. Today, insects are regularly consumed by approximately 2 billion people^25^ spread across 80% of the world’s populations^30^ in 130 countries^31^. Edible insects are gaining traction in North America and Europe, in addition to regions that traditionally practice entomophagy. The commercial industry was valued at 33 million USD in 2015, with future growth estimated at more than 40% by 2023^32^. Insects that have been eaten historically are generally considered safe for human consumption if properly processed like other animal products, although some people are allergic to insect proteins and chitin^33^. Generally, insects are a good source of bioavailable animal protein^33–36^ including all essential amino acids^26^, as well as B vitamins^35,37^, minerals^37,38^, and essential fatty acids^39^. Insects also contain relevant levels of crude fiber, most predominately in the form of chitin, derived from the exoskeleton^40^. A recent estimate of chitin and chitosan based on percent dry weight of whole ground crickets found values between 4.3–7.1% and 2.4–5.8%, respectively^41^.

Chitin (C_8_H_13_O_5_N)_n_) is a modified polysaccharide (poly-beta-1,4-*N*-acetylglucosamine) containing nitrogen with a structure analogous to indigestible cellulose; it is considered an insoluble fiber with potential prebiotic properties that could benefit human health by selectively promoting the growth of beneficial bacterial species in the intestines, though this relationship is not well understood. Chitin is the primary component of the exoskeleton, respiratory linings, digestive and excretory systems of arthropods^42^, and given the variation in insect anatomy chitin levels in common feeder insects vary widely^43^. Chitin has applications in health, drug delivery, agriculture, gene therapy, food technology, nano-technology, and bioenergy, among others^44^.

While the nutritional value of edible insects is widely documented^25,33,45^, other potentially beneficial properties of edible insects beyond nutrition have not been evaluated. To-date, no comprehensive clinical studies have investigated the impact of insect consumption on the human microbiome. Additionally, the health implications and the tolerability of insects, insect-based food, and insect-derived dietary fibers, including chitin, have not been assessed. The tolerability and safety of edible insects as a food and fiber source, as well as the effects of insect consumption on human microbiota, mucosal immunity, and other host parameters must be better understood in order to anticipate and optimize the effects of edible insects on human health for the 2 billion people that regularly eat them – as well as future consumers in the globally ascendant market for edible insects.

In this pilot study, we use a randomized, double blind, crossover diet intervention to evaluate the effect of consuming a commercially available 100% whole cricket powder (*Gryllodes sigillatus*) on gut microbiota composition in healthy adults. This study aims to determine if insect powder acts as a prebiotic, supporting growth of selective commensal bacterial species that confer health benefits. Additionally, we assessed safety and tolerability using comprehensive metabolic panels and gastrointestinal symptom questionnaires, several fecal metabolites, and markers of both systemic and local inflammation and immune function. The objective of this study was to gain baseline information regarding the impact of cricket consumption on gut microbiota in healthy adults, and to ascertain tolerability levels. Our findings enhance current understandings of benefits and risks of insect consumption and inform future research.

## Materials and Methods

### Participant Eligibility and Recruitment

Twenty healthy adults, aged 18–65 were recruited via flyer in Fort Collins, Colorado. Since no previous crossover trials have evaluated insect consumption impacts on microbiota, a sample size of 20, with balanced design and ten people per treatment, was selected for this pilot study using a 5% significance level (one-sided) to obtain 90% power. A crossover design was appropriate to address research questions and because intra-individual variances are lower than inter-individual variances. Eligibility was determined using an in-person eligibility screening questionnaire at an initial visit after which informed consent was obtained. Participants were excluded if they met any of the following criteria: (a) younger than 18 or older than 65, (b) BMI outside of the 18.5–29.9 range, (c) pregnant or breastfeeding, (d) use of antibiotics in the last 2 months, (e) regular use of prebiotics or probiotics, (f) any intestinal or metabolic disease, cancer, liver or kidney disease g) self-reported presence of food allergies, (h) unwillingness to limit alcohol consumption to 1–2 drinks per day, no more than 7 per week, or (i) current medication or dietary supplement use that may impact gut microbiota. These conditions are known to affect baseline microbiota populations. Additionally, only healthy volunteers were selected because standard care practices for people with various medical conditions would confound measured endpoints, including microbiota composition. This would include taking statins, metformin, NSAIDs, MAO inhibitors, and botanical supplements that target the GI tract or gut microbiota. Healthy individuals are more likely to comply with study requirements and experience fewer adverse events. To be eligible, participants had to confirm willingness to eat one prepared breakfast per day (treatment or control) at home for a total of 28 days (two treatment periods of 14 days each), attend three clinic visits, and provide three blood and stool samples.

### Study Design and Dietary Intervention

The study was conducted at Colorado State University between February and May 2017. The study protocol and documents were approved by the Institutional Review Board (IRB) for Human Subjects Research at Colorado State University, CSU protocol #16-6966 H and all participants provided written informed consent prior to beginning the study. All experiments were also performed in accordance with relevant guidelines and regulations. The study is also registered at clinicaltrials.gov as NCT03383341 on December 26, 2017.

The study was conducted as a randomized, double-blind, crossover trial, with two 14-day intervention periods and a 14-day washout period between treatments for a total duration of 42 days. Each study participant was randomly assigned to starting group, cohort 1 or 2, by the study coordinator. A laboratory volunteer that was not involved in study design or conduct assigned codes to treatment foods. Study personnel remained blinded to treatment assignment until after all data were collected and analyzed.

During each intervention period, study participants were provided with a breakfast that included a muffin and a dry breakfast shake mix, which they were instructed to combine with the milk or liquid of their choice and drink after shaking vigorously. The nutrient contents of the study breakfasts are outlined in Table 1. Participants were asked to return packaging and any uneaten portion of the foods to assess compliance.

**Table 1:** Estimated Nutrient Composition of Study Breakfasts.

Nutrient Composition Prepared Breakfasts (values per serving: 1 shake, 2 muffins)		
Nutrient	Control	Cricket
Energy (kcal)	495.26	569.34
Total fat (g)	12.75	18.12
Total protein (g)	9.31	21.67
Total carbohydrate (g)	88.36	81.34
Sugars (g)	46.72	48.65
Total fiber (g)	5.35	5.57

After enrollment, participants were randomly allocated to one of two sequential treatment arms. The first (Cohort 1) received the control breakfast meal for 14 days, followed by the cricket breakfast meal. Cohort 2 received the treatments in reverse order. Both had a washout period of 14-days between each intervention period. All meals were provided to volunteers in identical mylar bags labeled with an alphanumerical code unique to the treatment and participant.

**Figure 1:**
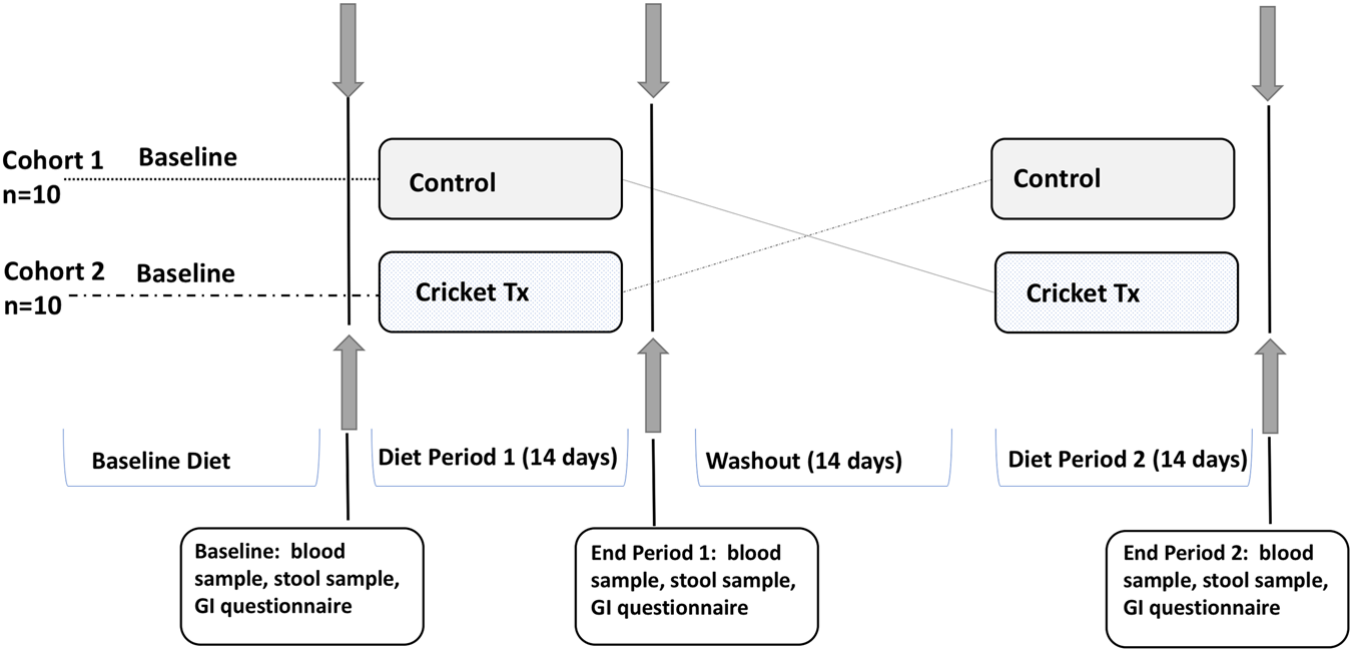
Study design. Cricket Tx = Breakfast with 25 g Cricket Powder; Control = Breakfast without Cricket Powder.

Subjects reported to the Human Performance Clinical Research Laboratory (HPCRL) at Colorado State University three times during the trial to provide a fasting blood sample, a stool sample, and to complete the gastrointestinal (GI) questionnaire. The first was at baseline (Day 0), before beginning the intervention. The second was after the first intervention period (Day 14), and the third was at the end after the second intervention period (Day 42). GI questionnaires were used to collect information on side effects from the intervention. The complete study design is outlined in Fig. 1.

### Dietary Intervention

Dried, roasted cricket powder was provided to the research team by Entomo Farms (Ontario, Canada). Participants received 14 prepared study breakfast meals that either included cricket powder (25 g/day) or that did not include cricket powder (control) at the beginning of each treatment period. They were asked to consume one prepared breakfast every day during the intervention periods but were able to consume their normal diet the rest of the day. The breakfasts included a pumpkin spice muffin (roughly equivalent to 80 grams) and one dry mix chocolate malt shake (Table 1). A 25 g/day cricket serving size was selected to provide approximately 15 grams protein, similar to many protein rich breakfast drinks, and as a feasible dose for incorporation into palatable foods. Nutrient composition of the cricket powder alone is shown in Table 2.

**Table 2:** Estimated Nutrient Composition of Cricket Powder per serving (25 g).

Nutrient Composition per serving (25 g) *Gryllodes sigillatus* cricket powder		
Nutrient		
Energy (kcal)	117.89	
	**Weight (g)**	**Percentage**
Total fat	6.00	24.00%
Total protein	14.78	59.12%
Total carbohydrate	2.10	8.40%
Sugars	0.13	
Total fiber	2.12	8.48%
Soluble	0.29	
Insoluble	1.83	

Both soluble and insoluble fiber were determined using Official Method 991.43 of the AOAC^86^. Estimate does not include ash, vitamins, minerals.

The control and the cricket intervention breakfasts (muffins and shake) were similarly matched in their macro- and micronutrient content, but the control did not contain any cricket powder. In both the control and cricket breakfast shakes, chocolate malt was used as a strong flavoring, and a small amount of instant pudding mix added to keep insoluble ingredients in suspension. The meals were identical in ingredients, with the exception of the following amendments made to the control breakfasts: purple cornmeal (2 tbsp.) was added to the smoothie to mimic the texture of the cricket powder, and (~0.27 tbsp./muffin) cocoa powder was added to the control muffin to mimic the color of the cricket powder and supply dietary fiber. Like chitin, cocoa powder contains insoluble dietary fiber, but it is not novel in the American diet. Cocoa powder used in these muffins was about 33% dietary fiber. The remainder of the participants’ diets was not controlled.

The nutrient content of dried, roasted cricket powder was determined by two commercial laboratories (Covance Laboratories, Madison, Wisconsin and Maxxam Analytics, Ontario, Canada). Total dietary fiber was estimated to be 2.12 grams per 25 grams of cricket powder, with about 87% of it composed of insoluble fiber. Insoluble chitin is considered the most common form of fiber in insects^25^. Each intervention breakfast shake contained 10 g of cricket powder, and the muffins contained 15 g for a total daily intake of 25 g.

### GI Questionnaire

Using a digestive health questionnaire developed by Metagenics (see Supplemental Materials; Fig. S1) participants self-reported feelings related to digestive health at baseline, after treatment period 1 and after treatment period 2. Participants were asked to reflect on the previous two weeks to gauge changes in digestive health over the course of the study.

### Blood Chemistry

Three blood samples (~10 mL) were collected from each participant by venipuncture after an overnight fast (12 ± 2 hours) at baseline (day 0), the end of intervention period 1 (day 14), and the end of intervention period 2 (day 42). Samples were collected in lithium heparin and ethylenediaminetetraacetic acid EDTA tubes. Plasma was collected by centrifugation from the EDTA tubes and stored at −80 °C prior to analyses of circulating inflammatory markers. Two-hundred microliters of lithium heparin whole blood was analyzed immediately using the Comprehensive Metabolic Panel (CMP) (Abaxis Global Diagnostics; Union City, CA) on a Piccolo Xpress Chemistry Analyzer (Abbott; Princeton, NJ). The CMP included assessment of blood levels of sodium (Na+ mmol/L), potassium (K+ mmol/L), carbon dioxide (tCO_2_ mmol/L), chloride (Cl^−^ mmol/L), glucose (GLU mg/dL), calcium (CA mg/dL), blood urea nitrogen (BUN mg/dL), creatine (CRE mg/dL), alkaline phosphatase (ALP U/L), alanine aminotransferase (ALT U/L), aspartate aminotransferase (AST U/L), bilirubin (T-BIL mg/dL), albumin (ALB g/dL), and total protein (TP g/dL).

### DNA Extraction and Sequencing

Fecal samples were self-collected using a stool sampling kit within 24 hours of scheduled clinic visits and delivered refrigerated or frozen to the clinic coordinator. Once returned to the clinic coordinator, samples were stored at −80 °C until analyzed. Stool samples were subsampled with sterile cotton swabs. Fecal DNA was extracted using FastDNA® KIT (MP Biomedical; Santa Ana, CA; cat#116540400) following manufacturer’s instructions and including additional wash steps. Quantification and dilution of isolated DNA PCR was pooled for library preparation. Sample DNA was stored at −20 °C prior to generation of sequencing libraries.

Sequencing libraries were constructed by PCR amplification of the V4 region of the 16s rRNA gene using primers 515F and 806R following the protocol for the Earth Microbiome Project (http://www.earthmicrobiome.org/protocols-and-standards/16s/). Amplicons were purified using AxyPrep Mag PCR clean-up beads (Axygen; Corning, NY) quantified with Quanti-iT PicoGreen,dsDNA Assay Kit (Invitrogen; Eugene, OR), and pooled in equimolar ratios prior to sequencing at the Colorado State University Genomics Core facility using a 2 × 250 MiSeq flow cell (Illumina, San Diego, CA)^46,47^.

### Microbiota Analysis

Paired-end sequence reads were concatenated and all combined 16s sequences were filtered, trimmed and processed with the DADA2 (R bioconductor package)^48^ implementation included in the open source bioinformatics tool myPhyloDB version 1.2.1 (www.myphylodb.org/). Briefly, all primers were removed from each sequence using the open source Python program Cutadapt^49^ and sequence variants were inferred using the default pipeline in DADA2. Each sequence variant identified in DADA2 was classified to the closest reference sequence contained in the Green Genes reference database (Vers. 13_5_99) using the usearch_global option (minimum identity of 97%) contained in the open source program VSEARCH^50^. ANCoVA and DiffAbund analyses were conducted in myPhyloDB^51^, and MicrobiomeAnalyst^52^ was used to calculate alpha diversity scores and Bray-Curtis distances. The raw sequencing data and associated metadata will be made available upon request.

#### Changes in Microbial Metabolism (SCFAs and Bile Acids)

Frozen fecal samples were extracted for short chain fatty acids (SCFAs) using acidified water (pH 2.5) containing 5 mM ethylbutyric acid as an internal standard. Samples in acid water were vortexed for 5 minutes, sonicated for 30 minutes and centrifuged (10,000 RPM /10 mins) to remove particulate matter. Supernatant was collected and centrifuged again (10,000 RPM /10 mins) and 100 μl was transferred to a glass insert bottle and analyzed on a GC-FID (Agilent 6890 Plus GC Series, Aglient 7683 Injector series, GC Column: TG-WAXMS A 30mx 0.25 mm × 0.25um) using the GC OpenLab program. Samples were normalized to the internal standard, ethyl butyric acid, and quantified using standard curves generated from dilutions of commercial stocks of acetate, propionate, and butyrate.

Bile acids were quantified using the following methods. Stool samples (25 mg) were homogenized in 500 μL of NH_4_OH, with 5 μL internal standards glycodeoxycholic acid d-4, deoxycholic acid d-4, and taurocholic acid d-5. The mixture was vortexed and incubated at 60 °C for 1 hour followed by sonication for 30 minutes. One mL of HPLC grade water was added and incubated at −80 °C overnight. Samples were centrifuged at 4 °C at 10,000 rpm for 30 minutes and the clear supernatant was transferred to vials for Ultrahigh Pressure Liquid Chromatography-Mass Spectrometry (UPLC-MS) analysis.

Analysis was performed on a Waters Acquity UPLC coupled to a Xevo TQ-S triple quadrupole mass spectrometer (Millford, MA, USA), as described previously^53^. Chromatographic separations occurred on a Waters HSS T3 stationary phase column (1 × 100 mm, 1.8 µM). The mobile phases were 2 mM ammonium hydroxide (A) and methanol and water with 0.1% formic acid (B). The samples were held at 4 °C and column at 70 °C. The analytical gradient was carried out as follows: At 0 min, 0.1% B; time 0.5 min, 0.1% B; time 2 min, 30% B; time 15 min, 97% B; time 16 min, 97%B; time 16.5 min, 0.1% B; time 21 min, 0.1% B. Flow rate was 210 µL/min and injection volume was 2 µL. The mass spectrometry was operated in selected reaction monitoring (SRM) mode. Inter-channel delay was set to 3 ms and the MS was operated in both negative and positive ionization modes with capillary voltage at 2.1 and 3.2 kV. Source temperature was 150 °C and desolvation temperature was 500 °C with a gas flow rate of 1000 L/hr, cone gas flow 150 L/hr, and collision gas flow 0.2 mL/min. Nebulizer pressure was 7 Bar and argon was used as the collision gas. Waters TargetLynx software was used for peak integration.

#### Fecal Triglycerides

Fecal triglycerides were assessed using the Triglycerides Assay Kit (Cayman Chemicals, Ann Arbor, MI). Briefly, 75 mg of homogenized fecal sample was suspended in 1xNP40 reagent containing protease inhibitors. Samples were centrifuged at 4 °C for 10 minutes at 10,000 rpm. Supernatant was diluted 1:5 with 1xNP40 and absorbance at 530–550 nm was measured after incubation for 15 min at room temperature. Triglyceride quantity was determined by fitting to standard curves.

#### Measures of Inflammation (Fecal secretory immunoglobulin A and cytokine analyses)

To assess changes in oral tolerance and mucosal immunity, fecal secretory immunoglobulin A (sIgA) was analyzed using the Human Secretory IgA ELISA Assay Kit (Eagle Biosciences, Amherst, NH) following manufacturer’s instructions.

System inflammation was assessed by measuring plasma levels of GM-CSF, IFNα, IL-1α, IL-2, IL-4, IL-5, IL-6, IL-7, IL-8, IL-10, IL-12 (p70), IL-13, and TNF-α using the Milliplex MAP Human High Sensitivity T Cell panel (Millipore Sigma, Burlington, MA). All samples were processed according to the manufacturers’ protocols and analyzed on a Luminex instrument (LX200; Millipore, Austin, TX).

### Statistical Analysis

To estimate the effect of the cricket diet on the various outcome measures, separate linear mixed models were fit for each outcome of interest using the restricted maximum likelihood (REML) criterion in the lme4 package (V 1.1–13) in R (V 3.3.2)^54^. Each model was adjusted for treatment group, baseline measure and period of treatment while the study participant was modeled as a random effect^55^. Following the recommendations of Senn *et al*. in regards to crossover designs, the washout period was assumed to be sufficient (no non-negligible carry-over effects) and an interaction term between period and treatment was omitted from all models^56^. After the models were fit to the data, semi-parametric, bootstrap 95% confidence intervals (4,000 iterations) were estimated for each model parameter and approximate p-values were calculated using the Satterthwaite approximation as implemented in the lmerTest package (V 2.0–36)^57^. Given that this study was intended as a pilot, no multiple-testing corrections were applied to model parameter p-values.

A graphical analysis of the residuals for 47 of the 55 models revealed no noteworthy violations of the assumptions of a linear mixed model. For eight of the models however (sIgA, fecal triglycerides, IL-13, ursodeoxycholic acid, 3a_6b_7b-trihydroxycholenoic acid, glycochenodeoxycholic acid, tarurodeoxzycholic acid and taruocholic acid) heteroscedasticity in the error variance could be seen in the residuals, hence the models were refit on the natural logarithm scale (where baseline measure was also log-transformed). Because some participants had sIgA values measured at zero, a small constant (c = 0.1) was added to measured values to allow for the log-transformation. For completeness, both model summaries (adjusted and unadjusted) for each of these outcomes are included in the appendix. No apparent model violations were found in graphical analysis of the residuals for the updated models.

Microbiota data were normalized using Laplace smoothing^58^ followed by subsampling with replacement (rarefaction (keep) command)^51^. Two individuals were omitted from the microbiota analyses due to a low number of sequence reads obtained for one or more of their time points. Data were rarefied to 24,150 sequence reads using 100 iterations. An Analysis of Covariance (ANCOVA) model was used to assess taxonomic differences across treatment groups, and genewise negative binomial GLM (DiffAbund; adjusted p-value for statistical significance was set as q < 0.1) was used to determine differential analysis of taxa relative abundance between treatments. Measures of alpha (CHAO1 estimates, Shannon diversity index) and beta diversity (Bray-Curtis distances) were statistically analyzed using non-parametric Kruskal-Wallis tests. All descriptive statistics were calculated using R (V 3.3.3).

### Data Availability Statement

The datasets generated during and/or analyzed during the current study are provided in Supplemental Materials or are available from the corresponding author on reasonable request.

## Results

### Participant Retention and Baseline Characteristics

**Figure 2:**
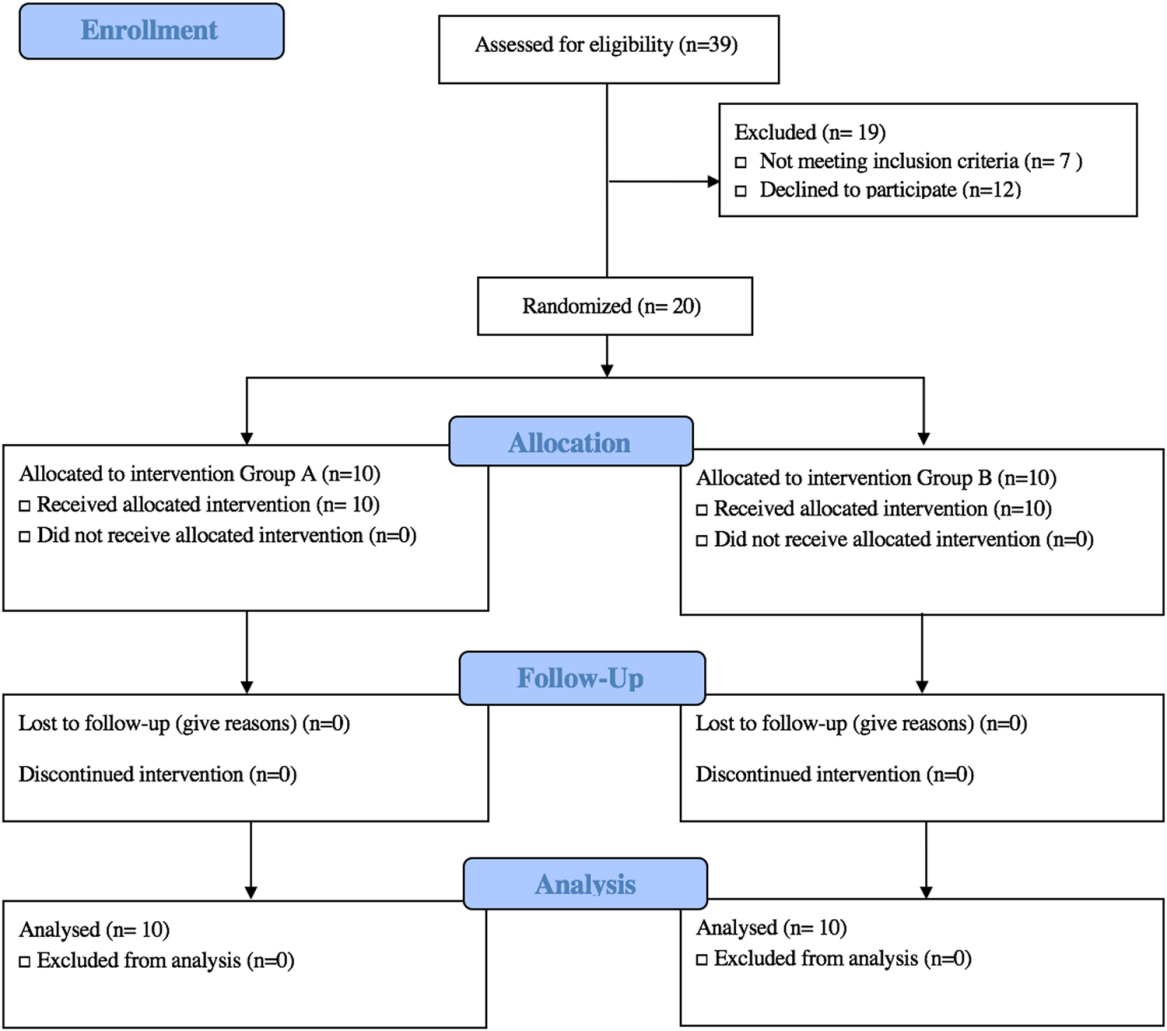
Consort Study Flow Diagram.

Twenty healthy adults, aged 20–48, with a body mass index (BMI) of 19.58–29.19 kg/m^2^ enrolled in the study, and all 20 completed the study. It should be noted that the general health and age of this population is not representative of the American population. Participants were younger, with lower average BMI. Ten participants were allocated to Cohort 1 and 10 to Cohort 2 (Fig. 1). One participant in Cohort 2 did not adhere to all the study requirements (~80% compliance), but was still included in data analysis based on the “intention-to-treat” principle^59^. Figure 2 diagrams the study flow. The average study participant age was 26.68 years, with an average body mass index (BMI) of 23.58. Table 3 includes the characteristics of participants at baseline. A comprehensive summary of participant measures is included the Supplemental Materials (Table S1).

**Table 3:** Participant Characteristics at Baseline Visit (mean +/− standard deviation).

Characteristic	Value
Age (years)	26.45 ± 6.33
Sex	
Male (%)	9 (45%)
Female (%)	11 (55%)
BMI (kg/m^2^)	23.39 ± 2.46
Fasting blood glucose (U/L)	89.32 ± 6.94

Note: values presented as the mean ± the standard deviation.

### Dietary Intervention

Participants were compliant with the dietary intervention during the two treatment periods. Since participants returned the Mylar bag containers with any unconsumed food to the study coordinator at their clinic visits, we were able to assess adherence to the study protocol. Consumption of 80% of more of study foods was considered full compliance. As mentioned above, only one participant in Cohort 2 failed to adhere to these requirements but was still included in data analysis.

### Documentation of Adverse Side-effects and GI Questionnaire

Responses from the GI questionnaire were pooled per instrument instructions according to health function for each treatment period. Health functions assessed included the colon, the small intestine and pancreas, generic GI inflammation, and overall gastric function. These results were run through a linear mixed effects model. No significant changes in GI function were reported across the study relative to baseline. Participants did not report any significant side-effects from cricket consumption. Results of the linear mixed effects model controlling for period effects and baseline responses are included in the Supplemental Materials (Table S2).

### Comprehensive Metabolic Panel

Blood chemistry analyses of Na, K, tCO_2_, Cl^−^, GLU, CA, CRE, ALT, AST, T-BIL, ALB, and TP showed that group averages fell within clinically normal reference ranges, and there were no significant differences throughout the study period. A slight period effect was observed with BUN (95% CI (0.55–2.61) p-val < 0.01), although mean values remained within normal clinical parameters (see Supplemental Materials Table S3). The cricket treatment was also associated with a slight increase in ALP (2.011 [95% CI (0.06–4.09) p-val < 0.05]) but the wide confidence interval suggests limited certainty. Regardless of this effect, most participants retained ALP enzyme values within the normal range (42–141 U/L) suggesting no pathological effects associated with cricket consumption. For those with ALP levels outside of this range, changes between the control and the cricket treatment were not significant relative to individual baseline level. Overall, no toxicity from cricket consumption was observed via blood panels.

### Changes in Microbial Metabolism (Fecal SCFAs and Bile Acids)

**Figure 3:**
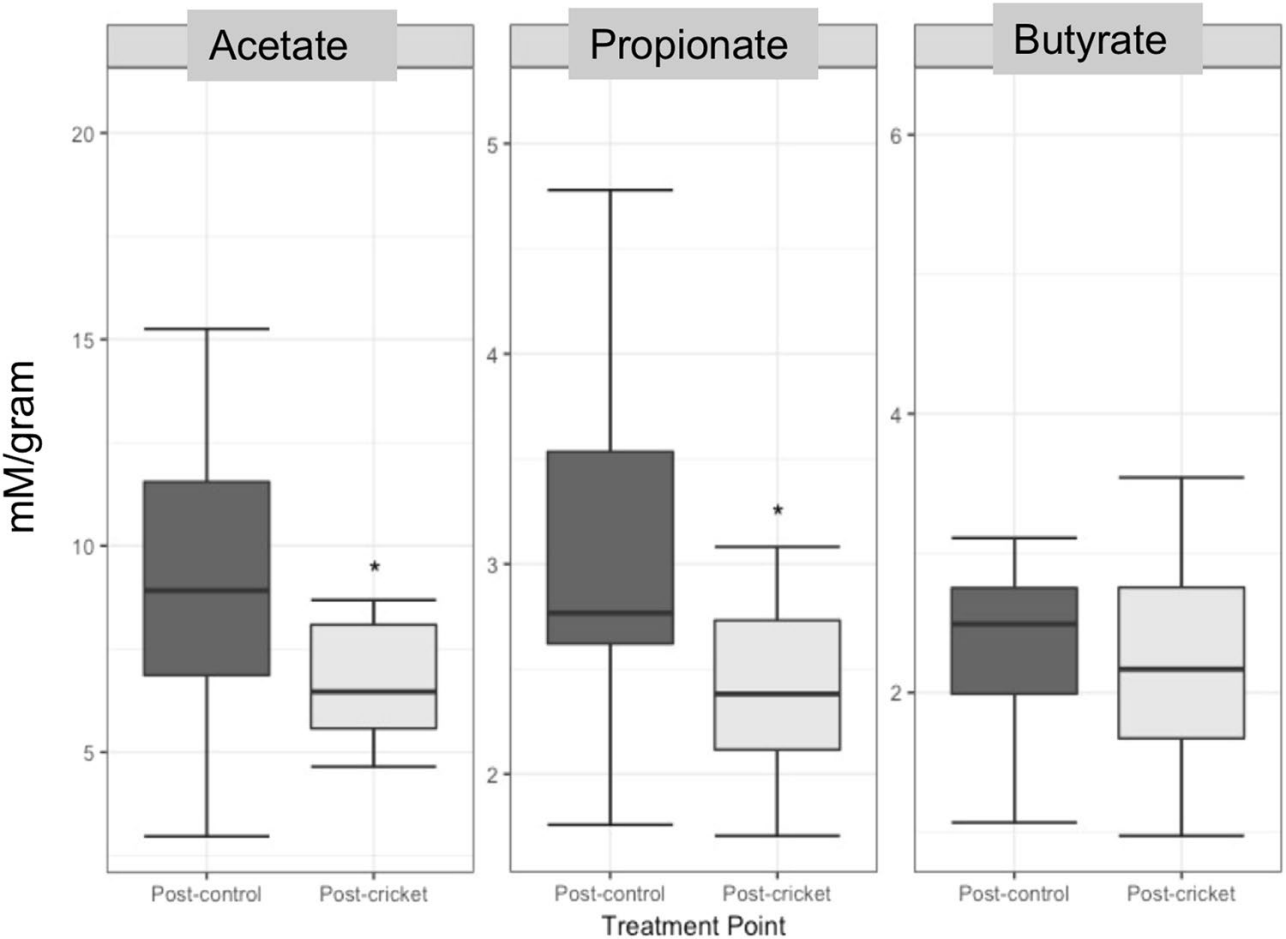
Boxplots of Average Excreted SCFAs across Treatment Periods (uM/g).

Fecal SCFAs are the end products of complex carbohydrate fermentation in the colon. The major SCFAs include butyrate, which is mainly utilized as an energy source by colonic epithelial cells, and acetate and propionate, which can modulate human metabolism through their role as signaling molecules. Changes were not observed in excreted butyrate across treatment periods (Fig. 3). However, acetate observed in the stool was reduced by an estimated 2.31 uM/g (95% CI (−4.02–0.05) p-val < 0.05) during the cricket diet. Similarly, cricket consumption was also associated with reduced propionate, estimated −0.58 uM/g (95% CI (−0.97–0.20) p-val < 0.01) as shown in Supplemental Materials (Table S4).

No significant changes in excreted bile acids or fecal triglycerides were observed using the model fit to the data.

### Microbiome Changes with Cricket Consumption

**Figure 4:**
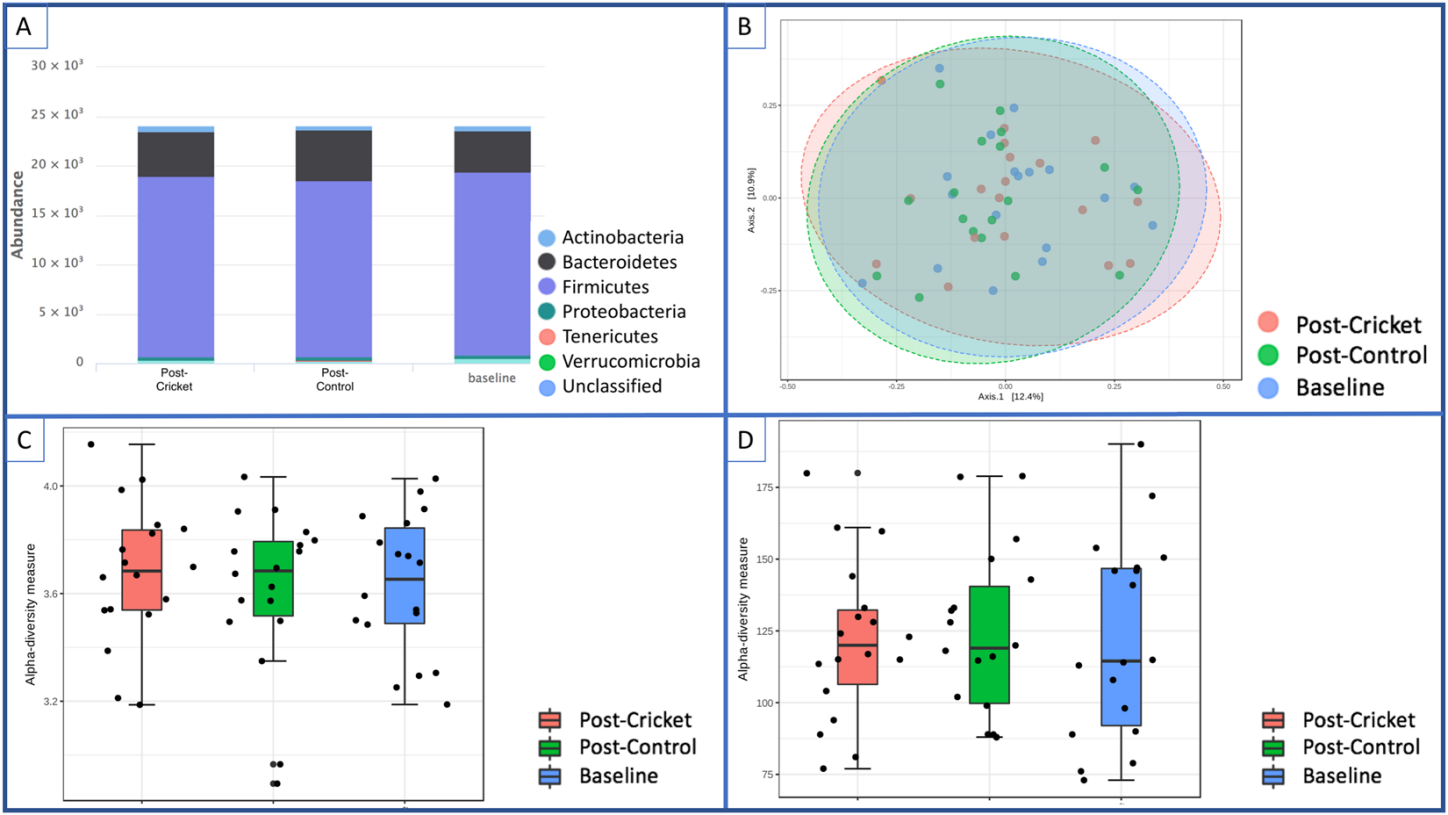
Changes in Participant Microbiota at Baseline, Post-Cricket, and Post-Contro. (**A**) Phyla-level bacterial composition of stool samples for all participants at baseline and post-interventions suggests global stability of the microbiota across treatments. (**B**) Principle Coordinates Analysis (PCoA) projecting Bray-Curtis distances. (**C**) Shannon diversity scores, and (**D**) Chao richness estimates further confirm global microbiota stability across diet treatments.

A total of 1,304,100 reads were used for microbiota analysis, and 855 sequence variants were detected. No significant changes in phyla-level microbiota composition were observed across the treatment groups (Fig. 4[A]). Bacteroidetes and Firmicutes made up ~90% of sequences at phyla level. There were also no significant differences in OTU richness or Shannon diversity scores. In general, inter-individual differences in gut microbiota composition were greater than treatment effects. Principle Coordinates Analysis (PCoA) using Bray-Curtis distances suggests that samples from an individual were more similar than samples between individuals, regardless of treatment group (Fig. 4(B)); however, levels of response varied with certain individuals displaying a large amount of variability between sample time points (i.e., Hop4, Hop7, Hop15 and Hop20) (see Supplemental Materials Fig. S2).

**Figure 5:**
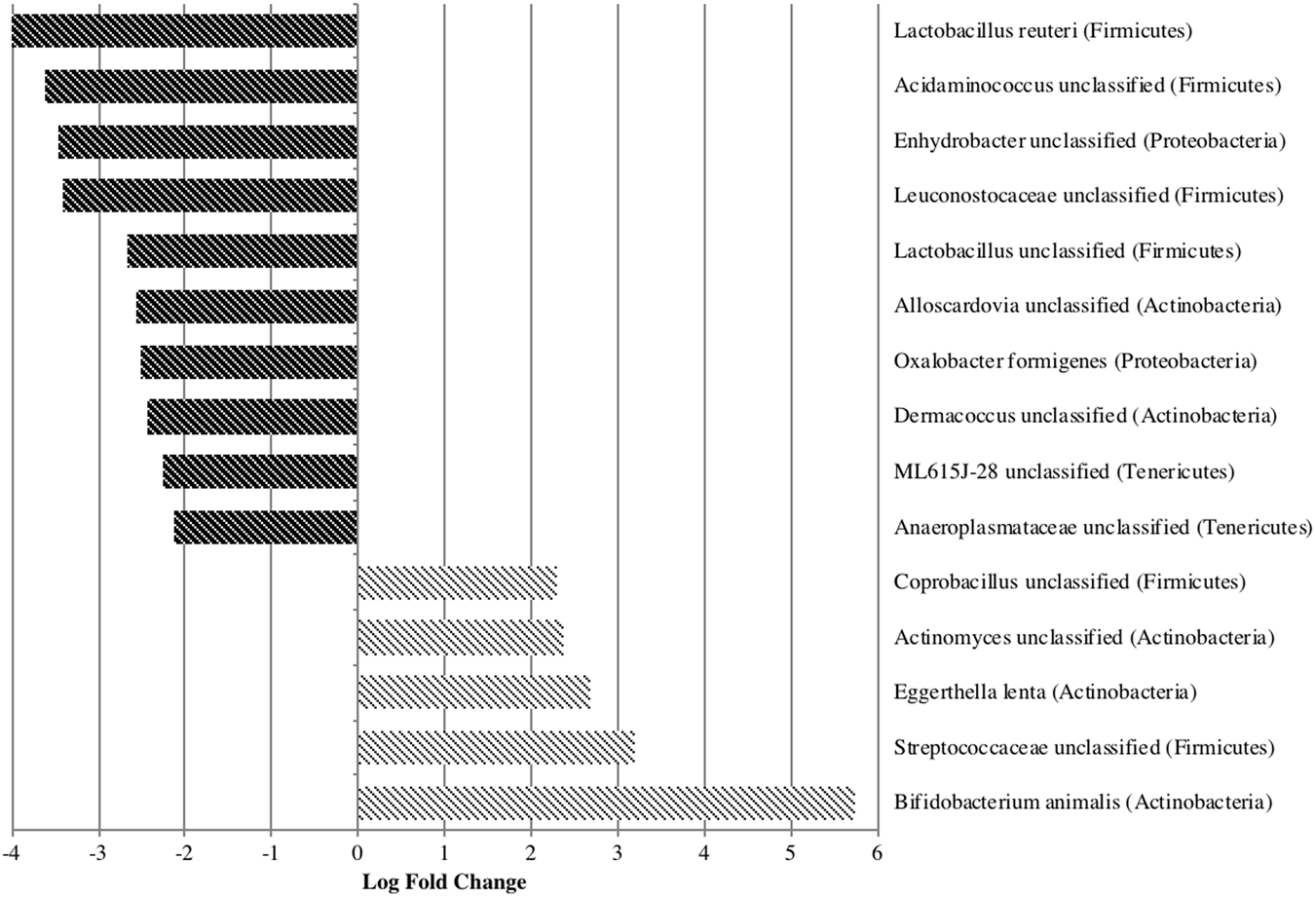
EdgeR analysis (negative binomial GLM) identified several taxa that significantly contributed (q < 0.1) to differences between post-cricket and control microbiota.

Pairwise comparisons of the species-level abundance using a negative binomial GLM model (edgeR) revealed that the fold-change of several taxa was significantly different (q < 0.1) between the treatment and control diet periods (Supplemental Materials Fig. S3). Five bacterial taxa significantly increased after cricket consumption, including three OTUs that correspond with members of the phylum Actinobacteria (Fig. 5). Actinobacteria are commonly found in the human gut^60^. Specifically, the probiotic species *Bifidobacterium animalis* increased by a log fold change of 5.7 on the cricket diet compared to the control diet.

In contrast, the probiotic species *Lactobacillus reuteri* and two other lactic acid-producing bacteria (LAB) were decreased by 3 to 4-fold relative to control after two weeks of cricket consumption. In addition to decreased LAB, bacteria in the genus *Acidaminococcus* were reduced more than 3-fold with cricket consumption.

### Mucosal Immunity and Systemic Inflammation

Fecal sIgA antibodies are an essential component of intestinal epithelial defense against pathogens and enteric toxins. It is a measure of tolerance, and major shifts in sIgA might indicate reduced mucosal immunity (low levels) or an inflamed gut (high levels). No changes between baseline, treatment, and control were observed for fecal sIgA. We observed high sIgA heterogeneity across participants. Most participants in this study retained normal stool levels of sIgA (510–2040 µg/ml) at baseline and after consuming both the control breakfast and the cricket breakfast (see Supplemental Materials Fig. S4).

Numerous circulating cytokines that mediate inflammatory responses are produced by T-cell populations. A Human T-cell panel of 13 cytokines/chemokines revealed only one significant change in a circulating cytokine in plasma from 10 randomly selected participants. Tumor Necrosis Factor alpha (TNF-α) was lower after cricket consumption compared to the control diet (−0.525 [95% CI (−0.93–0.12 p-val < 0.05)]. Using the model fit to the data, changes in other cytokines were not significant (see Supplemental Materials Tables S5 and S6).

## Discussion

The goal of this pilot cross-over study was to assess the tolerability and impact on the microbiota of consuming whole cricket powder. Findings suggest that consumption of 25 g of cricket (*G*. *sigillatus*) daily for 14 days is safe and does not yield adverse clinical outcomes. All 20 adult participants completed the study and self-reported consuming > 85% of the allotted study foods, suggesting that study protocol was achievable for volunteers. The study utilized a balanced, even crossover design (AB/BA), along with a 2-week washout period, to prevent sequence carryover effects. Previous studies have indicated that diet-induced changes to the microbiota can revert to baseline levels within 24 hours of cessation of intervention foods^14^.

Gastrointestinal symptom self-assessments and data from comprehensive metabolic panels suggest that cricket consumption was both safe and tolerable in our study population.

Cricket consumption during the study period did not dramatically disrupt the healthy adult microbiota. However, cricket intake was associated with an increase in abundance of five bacterial taxa, one of which most closely aligned to sequences of *Bifidobacterium animalis*. *B*. *animalis* is a Gram-positive, non-spore forming, lactic acid producing bacteria. It is one of the best studied probiotics, and a commercial strain, *B*. *animalis* subsp. *lactis* BB-12^®^ has been shown in clinical studies to improve gastrointestinal function, protect against diarrhea, reduce side effects of antibiotic treatment, and increase resistance to common respiratory infections^61^. Chitooligosaccharides, similar to those found in cricket, are reported to be bifidogenic at between 0.1–0.5% *in vitro*^62^. In culture, *B*. *adolescentis* can utilize N-acetylchitooligosaccharide as a carbon source^63^. To our knowledge, this is the first time a whole food containing chitin has demonstrated bifidogenic potential in healthy humans.

Pathogen inhibition is one of the primary mechanisms by which probiotics influence human health, along with enhancing gut barrier function. Probiotics also interact with the immune system. In assays, BB-12^®^ displayed antagonistic activity toward pathogens, including enteropathogenic microorganisms such as *E*. *coli* and *B*. *cerus*, demonstrating an ability to generate inhibitory zones^64^. BB-12^®^ also helps reduce cellular attachment of pathogens, as was demonstrated in a pig study where treatment of intestinal mucus with the probiotic significantly reduced adhesion of tested pathogens including *Salmonella*^65^. Moreover, studies have demonstrated that BB-12^®^ may be able to interact with immune cells and have an overall beneficial effect on immune system function^61,66,67^.

Cricket consumption was associated with suppression of *Acidaminococcus* among participants. Increased relative abundance of *Acidaminococcus* has been associated with linear growth deficits in infants from Malawi and Bangladesh^68^. While linear growth deficits are not relevant in the current study population, there may be important implications for cricket consumption in maternal and child health in low-resource settings. Another notable change in gut microbiota associated with cricket consumption included a 3 to 4-fold decreased abundance of *Lactobacillus spp*. (including *L*. *reuteri*) and *Leuconostoc*. Reduced abundance of certain LAB with cricket consumption could be related to substitutions made to breakfasts or potentially to the antimicrobial properties of chitin. LAB is common in yogurt, which is a typical breakfast food for many American consumers. We suspect that by fully substituting breakfasts for study participants, some observed decreases in LAB may be due to reduced ingestion of common LAB-containing foods. In addition, LAB are commonly consumed as supplemental probiotics. Although all participants were asked to stop consuming any probiotic supplements for at least one week prior to the start of the study and refrain from probiotic consumption throughout the study, it is possible that higher baseline levels might have reflected previous probiotic use. Future studies may consider collecting daily diet records in order to gain further understanding of how cricket consumption influences intake of other foods.

Moreover, chitooligosaccharides were previously shown to have potent inhibitory activity against *Lactobacillus*^69^, and chitosan, the deacetylated form of chitin, has antimicrobial activity against *Lactobacillus spp*. responsible for beer spoilage in breweries^70^. This suggests that treatment with chitin and chitosan may also exert effects on the microbiota through antimicrobial activity. Long-term impacts of cricket consumption on LAB require further investigation.

SCFAs serve as signaling molecules between microbiota and their human host, helping to regulate intermediate and peripheral metabolism. SCFAs are known to influence gut integrity, glucose homeostasis, lipid metabolism, appetite regulation, and immune function^71^. Fecal SCFAs levels are often reflective of dietary fiber intake and are generally thought of as a useful biomarker for gut microbiota activity and health^72,73^ even though 90–95% are typically absorbed in the intestines. This study assessed excreted SCFAs to measure changes in microbial metabolism. We observed small reductions in excreted acetate and propionate with cricket consumption, although butyrate levels were unchanged. These reductions suggest that the full breakfast substitution could have removed some crucial soluble fibers from the diet (e.g., oatmeal) which generate these SCFAs through fermentation. Alternatively, while chitin is found in shellfish and fungus, whole crickets are a novel food and fiber source in the diet of this target population. Thus, it is possible that participants’ gut microbes were not fully equipped to digest insect fibers. Previous research has shown that different consumer populations exhibit variable microbial activity when exposed to the same diet. For example, a recent study demonstrated that trimethylamine production from dietary carnitine found in red meat was a result of gut microbial metabolism in the gut of omnivores, whereas a vegetarian/vegan population consuming dietary carnitine lacked the necessary microbes for this conversion^74^. Another study has suggested an extinction of microbes capable of metabolizing microbiota-accessible carbohydrates in populations that consume a typical western diet^75^, supporting the hypothesis that an unadapted microbiota may not be able to utilize novel fiber sources like chitin.

Chitin may also be effective in the control of lipid absorption in the intestines. Chitin intake at 5% by weight of total food intake in rats led to lower total plasma cholesterol and lower LDL-cholesterol, as well as to higher excretion of triglycerides in feces^76^. However, we did not examine plasma lipids due to the short duration of the study, and our results did not indicate any changes in lipid metabolism as indicated by fecal triglyceride and bile acid secretion. These results are in concurrence with a pilot test of chitosan consumption in healthy adult men on a high fat diet, where they did not observe any effects on fat absorption^77^.

Finally, we examined parameters of mucosal immunity and systemic inflammation. Cricket consumption did not significantly alter sIgA, a measure of mucosal immunity. Low sIgA (<510 µg/ml) is associated with compromised mucosal immunity, which could be related to a mild GI condition or stress while high sIgA (>2000 µg/ml) typically occurs with pathogen exposure^78^. There was a high amount of variability in baseline sIgA and some individuals were either above or below the normal clinical range. However, a lack of treatment-related effects was likely due to the fact that our participants were primarily young, healthy normal weight adults. Since the majority of study participants were college students, it is reasonable to assume that stress-related factors could account for the baseline variability seen in this population.

Examination of 13 chemokines/cytokines revealed that TNF-α in plasma was reduced with cricket consumption relative to the control diet. TNF-α is a pro-inflammatory cytokine, and increased levels have been associated with intestinal inflammation and several inflammatory gut conditions^79^. TNF-α is upregulated downstream of Toll-like receptor 4 (TLR4) activation by bacterial lipopolysaccharides (LPS), and reduced levels may be due to improvements in gut barrier function, resulting in less LPS translocation from the intestinal lumen into systemic circulation^80^. Indeed, *Bifidobacterium* supplementation has been shown to modulate improvements in barrier function^81^. Additionally, clinically low ALP is associated with poor nutrient absorption, and some participants fell below the clinical reference range (42–141 U/L), although average ALP values were within range across treatments, suggesting increases were not pathological. Thus, our observation of significantly increased ALP combined with reduced circulating TNF-α may be indicative of an overall improvement in intestinal homeostasis. Moreover, the influence of diet on production of inflammatory cytokines like TNF-α has been linked with a number of important health endpoints including cancer incidence^82^, cardiovascular disease^83^ and major depression^84^. Whether consumption of edible insects should be recommended as a strategy to improve overall diet quality and positively influence these health endpoints requires additional empirical evidence.

This study provides preliminary evidence that edible cricket consumption at dose of 25 g per day is safe and may alter specific populations within the gut microbiota. Since participants’ diets outside of the provided breakfasts were not controlled, we cannot confirm that observed changes in measured outcomes were due exclusively to cricket consumption. However, cricket represents a novel food for American populations, and participants were instructed to continue to eat their regular diet during the study period. Additionally, we were unable to assess the dose response of cricket consumption in this study. More research, including clinical trials with greater sample sizes and completely controlled dietary interventions, is needed to assess the precise impact of cricket consumption on the healthy gut microbiota. Moreover, nutritional impacts of insects, specifically due to chitin, warrant further exploration; it is possible that dietary chitin interacts with nutrient absorption.

In the current study, it is impossible to conclude that the observed changes were due to chitin rather than to other insect components as we used a whole ground cricket powder in our study population. While cricket chitin may function as a prebiotic, dietary intervention studies that test chitin in isolation are needed to confirm prebiotic effects. Regardless of the function of specific cricket components, whole cricket powder consumption did not have any apparent negative effects on gut health in our healthy population and may have exerted some benefits. These findings suggest that evaluation of the impact of insect consumption on individuals with environmental enteropathy is warranted. The prevalence of entomophagy across parts of southeast Asia and sub-Saharan Africa that currently face high rates of malnutrition and food insecurity raises an interesting question regarding the health benefits of insect consumption, as long-term dietary patterns are the primary determinants of taxonomic and functional structure of gut microbiota^85^.

## Conclusion

To our knowledge, this is the first study of its kind to evaluate the impact of edible cricket consumption on the human gut microbiota. We provide evidence that cricket supplementation selectively changes the gut microbial and metabolite environment, but it does not dramatically shift the global gut microbiota after a 14-day dietary intervention. Similarly, we demonstrate that cricket consumption was safe over the study period and was not associated with major GI side-effects. These findings support the need for future research to evaluate the health impacts of edible insects, *beyond* their nutritional value, on human microbiota. This may be particularly relevant in populations at risk for malnutrition or environmental enteropathy. Further scrutiny is justified to determine how these observed changes translate into health outcomes. Studies with greater sample sizes, longer durations, and variable doses of insect consumption are warranted. It would also be prudent to evaluate the impact of isolated insect chitin on the gut, and to compare these findings with other studies that have investigated chitin and chitin derivatives from other sources. Studies enforcing a fully controlled dietary intervention are needed to elucidate the precise impact of cricket powder on microbiota without other confounding dietary factors. Lastly, we propose epidemiological studies of entomophagy among diverse populations that already consume insects to measure population level effects of insect consumption on the microbiome.

## Electronic supplementary material

Supplementary Materials
