# Supplementary material for: Impact of Edible Cricket Consumption on Gut Microbiota in Healthy Adults, a Double-blind, Randomized Crossover Trial
Source: Sci Rep. 2018 Jul 17;8:10762. doi: 10.1038/s41598-018-29032-2 (PMC6050247; doi:10.1038/s41598-018-29032-2)
Supplement: Supplementary file 1 — Supplementary Materials [file 41598_2018_29032_MOESM1_ESM.pdf]

What's Hopping: Impact of Edible Cricket Consumption on Gut Microbiota in Healthy Adults, a Double-blind, Randomized Crossover Trial

Valerie J. Stull,[1*] Elijah Finer,[2] Rachel S. Bergmans,[3] Hallie P. Febvre,[2] Colin Longhurst,[4] Daniel K. Manter,[5] Jonathan A. Patz,[1] and Tiffany L. Weir[2]

Supplemental Materials

Figure S1 – Digestive Health Questionnaire (see below)

Table S1 – All Participant Raw Outcome Data Across Treatments (excluding Cytokines)

Study Code (Participant)	Study Arm	Treatment	Period	Na+ mmol/L	K+ mmol/L	tCO2 mmol/L	CL mmol/L	GLU mg/dL	CA mg/dL	BUN mg/DL	CRE mg/dL	ALP U/L	ALT U/L	AST U/L	T-BIL mg/dL	ALB g/dL	TP g/dL	Acetate uM/g	Propiote uM/g	Butyrate uM/g	sIga ug/ml	Fecal Triglycerides mg/100mg
HOP-01	A	Control	1	140	4.5	23	109	72	9.6	12	1	69	30	44	1.6	4	6.8	8.8294	2.5676	2.2829	2192.6136	8.5686
		Cricket	2	145	3.6	25	105	81	9.6	18	0.8	79	21	36	1.2	3.9	6.8	8.0020	2.3057	3.5422	954.3478	3.8627
		Baseline	0	145	4.3	24	109	74	9.8	20	1	66	28	47	1.5	4.1	7	11.5184	2.3763	4.2731	2560.0094	10.5294
HOP-02	A	Control	1	140	4.2	25	108	81	9.8	14	1.1	46	19	21	0.6	4.2	7.6	2.9648	1.7586	1.1546	7894.1213	2.4902
		Cricket	2	142	4	27	100	93	9.7	21	1.1	47	16	28	0.6	4.3	7.8	5.8030	2.7750	2.1609	2760.7955	5.0392
		Baseline	0	140	4.7	26	107	86	9.7	17	1.1	35	21	21	0.9	4.4	7.6	18.0422	4.4293	4.0677	4647.4798	3.0784
HOP-03	A	Control	1	142	4.1	26	105	84	9.8	12	0.7	55	23	35	1	4.1	7.8	9.0037	3.0246	3.1090	1849.3371	3.0784
		Cricket	2	141	3.9	26	105	89	9.6	15	0.9	59	19	33	0.8	4.2	7.7	6.7520	3.0814	2.4436	6756.4815	7.7843
		Baseline	0	145	4.2	27	105	88	9.6	13	1.3	68	22	42	1.3	4.2	8.3	22.4331	6.5485	5.0161	1879.3527	5.6275
HOP-04	A	Control	1	145	4.1	26	105	85	9.5	9	0.7	73	25	25	0.5	3.8	7.6	11.4940	3.1479	2.9509	2045.0195	3.8627
		Cricket	2	140	4.1	21	105	81	9.4	10	0.5	77	19	29	0.8	4	7.7	8.6862	2.8645	2.7944	2647.1591	3.8627
		Baseline	0	142	3.7	20	104	89	9.4	9	0.7	82	21	33	0.9	4	8.3	8.0649	3.2118	1.6850	1228.1250	13.0784
HOP-05	A	Control	1	140	3.9	21	108	87	8.8	10	0.8	35	33	48	0.5	3.5	7.3	15.2514	4.7787	5.2281	1915.6250	5.6275
		Cricket	2	139	4	23	103	93	9.5	12	0.8	42	33	43	0.4	3.7	7.6	14.9655	5.0037	6.2200	7724.5571	8.9608
		Baseline	0	136	4.6	23	111	90	8.5	12	0.6	35	28	37	0.6	3.5	7.3	26.3903	7.1148	6.9794	2890.4891	2.6863
HOP-06	A	Control	1	131		16	105	104	9.2	9	0.6	33	28	54	0.8	4.1	6.6	8.2400	2.7574	2.0813	1459.8958	5.4314
		Cricket	2	136	4.2	26	100	94	9	13	0.7	40	24	32	1.2	3.7	6.6	4.6866	1.7061	1.5946	340.1042	3.8627
		Baseline	0	136	4.7	23	102	104	9.2	10	0.7	39	42	46	1.3	4	7.1	17.0702	3.8631	6.5383	0.0000	7.1961
HOP-07	A	Control	1	145	4.5	27	105	94	9.7	16	1.1	50	23	24	1	4.1	7.2	6.9813	3.4143	2.0575	571.3362	9.1569
		Cricket	2	140	4.1	27	104	92	9.2	15	1.1	60	32	34	1	3.9	7	8.4481	2.4788	2.1745	3379.6196	4.4510
		Baseline	0	145	4.4	28	104	85	9.5	18	0.9	49	22	25	1.1	4.2	7.1	9.1952	2.7309	2.1779	3516.6045	9.7451
HOP-08	A	Control	1	139	3.7	25	103	90	9.5	9	0.9	31	18	25	0.9	4	7.8	20.6920	4.1532	2.5154	27097.9167	2.8824
		Cricket	2	143	4.6	26	102	86	9.8	11	0.9	41	21	29	0.6	3.9	8.1	5.2816	2.1563	1.5534	1760.9069	3.2745

		Baseline	0	137	3.5	23	104	94	10.1	8	0.9	35	21	23	0.9	4.5	8.8	22.3326	8.2921	5.2825	13106.2500	2.8824
HOP-09	B	Cricket	1	140	3.9	26	106	81	9.3	18	1	28	26	35	1.2	3.8	6.9	11.7164	2.6886	2.6845	1750.6944	7.9804
		Control	2	145	3.9	23	105	73	9.4	17	0.9	30	30	33	0.9	4.3	7.2	8.3503	2.7182	3.1816	2292.6630	3.2745
		Baseline	0	139	3.9	24	106	80	9.5	16	0.8	29	22	38	0.8	4.2	6.7	16.9873	3.1844	4.9099	5783.4746	3.0784
HOP-10	A	Control	1	140	3.9	24	104	102	9.3	11	1	49	27	32	1.8	4	7.5	10.5400	3.8954	2.4870	1335.7215	4.2549
		Cricket	2	142	3.5	26	104	87	9.4	13	1.3	56	18	35	1.8	3.9	7.3	7.8945	2.5663	1.6821	2309.0653	6.8039
		Baseline	0	139	4	25	104	94	9.6	10	1	52	20	38	2.1	4	8	15.2273	5.6326	3.2044	2105.5201	7.7843
HOP-11	B	Cricket	1	137	4.5	26	100	92	10.1	15	1	55	25	29	1	4.1	8.1	6.1034	3.0261	1.5764		3.2745
		Control	2	140	3.7	27	103	94	9.5	12	0.9	49	31	33	1.6	4.4	8	7.3071	1.7821	2.4169	6627.5884	4.2549
		Baseline	0	144	4	25	106	89	9.9	13	1.1	71	23	36	0.8	4.2	8.3	18.0803	3.5470	5.4745	1661.8702	5.6275
HOP-12	B	Cricket	1	140	4	21	104	96	9.7	13	0.8	61	23	25	0.8	3.5	7.2	4.3757	1.8410	1.0674	157.8125	43.4706
		Control	2	137	3.7	21	107	87	9.4	9	1	63	21	27	0.5	3.6	7.7	5.5226	2.2172	1.7276	123.5950	3.8627
		Baseline	0															8.0203	2.5392	1.3567	996.1538	13.0784
HOP-13	B	Cricket	1	143	4	26	106	88	8.6	13	0.8	35	18	25	1.6	3.2	5.9	9.5755	2.6986	2.5026	1837.5000	2.6863
		Control	2	139	4.2	21	114	84	9	14	1	33	21	22	0.8	3.3	5.8	6.4452	1.7635	1.8021	1960.8553	12.6863
		Baseline	0	142	3.6	26	106	88	8.4	12	0.8	40	18	33	1.5	3.1	5.6	19.9844	3.7507	5.4708	2802.5735	2.6863
HOP-14	A	Control	1	141	4	23	104	88	8.8	15	1	57	26	30	1.2	4.1	7.4	8.2330	2.6385	2.0977	819.5076	3.8627
		Cricket	2	139	3.7	25	106	94	9.1	13	0.7	65	23	23	1.1	3.7	7.4	5.8371	1.9932	1.6712	3968.1818	6.8039
		Baseline	0	138	3.5	24	105	92	9.3	10	0.9	64	24	31	1.6	4.4	7.7	7.5893	2.7537	2.6036	5590.2902	5.6275
HOP-15	B	Cricket	1	140	3.9	20	104	91	9.6	14	0.7	38	22	31	0.8	3.9	8	5.6003	2.3004	1.6008	1876.5625	5.2353
		Control	2	138	4	24	105	77	9.4	13	0.9	33	19	30	0.7	3.8	7.7	4.6511	1.9195	1.1982	4146.7831	4.2549
		Baseline	0	136	3.8	18	101	81	9.5	14	0.8	38	24	34	0.7	4	8	16.9486	4.4727	6.0211	1314.6161	7.1961
HOP-16	B	Cricket	1	141				94	9	11	0.9	70	24	28	0.8	4	7	12.8169	5.1879	4.6037	1148.9407	26.0196
		Control	2		4.4	25	105	90	9.4	12	1.1	55	39	41	1.1	4.1	7	5.5918	2.4580	2.5620	0.0000	6.0196
		Baseline	0	143	4.1	24	107	89	9.5	10	0.8	64	28	33	0.8	3.8	7.3	13.9166	5.4759	6.0758	1042.6630	9.7451
HOP-17	B	Cricket	1	139	3.8	21	104	91	9.7	12	0.5	48	18	25	1	3.9	7.4	6.5102	2.3117	1.7918	2921.5601	8.1765
		Control	2	139	3.7	24	102	81	9.4	11	0.7	51	16	26	0.8	3.9	7.1	6.4850	2.2120	0.9719	5291.4474	3.8627
		Baseline	0	142	4.3	24	105	98	9.6	11	0.8	48	19	29	0.8	4.2	8.1	6.0911	2.3635	1.6756	12037.5584	2.8824

HOP-18	B	Cricket	1	143				96	9.5	14	1	46	15	25	0.6	4	7.4	9.3659	2.6975	2.6375	384.6491	4.2549
		Control	2		4.9	25	108	92	9.7	15	1.2	44	20	33	0.6	3.8	7.4	6.0807	2.5948	2.7407	3148.8839	2.6863
		Baseline	0	143	4.9	26	103	87	9.7	14	0.9	42	25	26	0.7	3.8	7.4	12.0446	4.2132	3.6354	4106.8452	7.7843
HOP-19	B	Cricket	1	138	4	27	105	94	9.4	15	1	89	51	53	0.8	3.9	7	13.2316	5.1151	5.6520	1525.0000	3.8627
		Control	2	139	4	27	106	94	9.4	13	1.1	89	48	37	0.6	3.7	6.6	11.9889	4.3729	4.3849	1081.2500	
		Baseline	0	142	4	24	106	99	9.6	16	1.3	86	37	45	0.7	3.8	6.9	30.2302	9.4166	11.1285	0.0000	5.6275
HOP-20	B	Cricket	1	139	4	30	101	98	9.5	14	1.3	88	35	32	1.3	4.3	7.4	8.2269	2.7751	2.4969	1853.4439	2.8824
		Control	2	141	3.9	28	105	93	9.1	12	1.3	88	24	31	1.5	4.3	7.1	4.8628	2.1711	1.6682	1731.2500	
		Baseline	0	142	4.2	26	106	90	9.4	15	1.1	84	35	42	0.6	4	7.1	9.9266	3.2821	2.6120	1678.8086	4.6471

Table S2 - Linear Mixed Effects Model of GI Indicators

Test	Coefficient	$\hat{\beta}$	SE ($\hat{\beta}$)	95% CI	p-value
Colon	(Intercept)	0.3683	0.4477	(-0.505, 1.246)	0.416
	Baseline	0.2726	0.1913	(-0.096, 0.638)	0.171
	Treatment	0.5000	0.4384	(-0.369, 1.359)	0.269
	Period	-0.3000	0.4384	(-1.145, 0.553)	0.503
Intestine	(Intercept)	1.0729	0.8532	(-0.603, 2.687)	0.217
	Baseline	0.6004	0.1055	(0.388, 0.807)	2.13e-05
	Treatment	0.4500	0.7167	(-0.940, 1.848)	0.538
	Period	-1.0500	0.7167	(-2.460, 0.334)	0.160
Inflammation	(Intercept)	0.3388	0.6283	(-0.884, 1.555)	0.59327
	Baseline	0.4255	0.1378	(0.149, 0.695)	0.00633
	Treatment	0.1500	0.5336	(-0.845, 1.192)	0.78183
	Period	0.5500	0.5336	(-0.496, 1.634)	0.31631
Gastric Function	(Intercept)	0.6408	0.5944	(-0.549, 1.807)	0.2905
	Baseline	0.4780	0.2276	(0.045, 0.942)	0.0501
	Treatment	0.4500	0.3905	(-0.339, 1.249)	0.2643
	Period	-0.5500	0.3905	(-1.3023, 0.225)	0.1760

Table S3 - Mixed Linear Effects Model Results of Comprehensive Metabolic Panels

Test	Coefficient	$\hat{\beta}$	SE ($\hat{\beta}$)	95% CI	p-value
Sodium (mmol/L)	(Intercept)<br>Baseline<br>Treatment<br>Period	93.9825<br>0.3270<br>-0.0190<br>0.4150	21.4695<br>0.1525<br>0.8226<br>0.8226	(52.081, 134.732)<br>(0.0367, 0.627)<br>(-1.685, 1.579)<br>(-1.188, 2.043)	0.000425<br>0.046418<br>0.981891<br>0.617048
Potassium (mmol/L)	(Intercept)<br>Baseline<br>Treatment<br>Period	3.3452<br>0.1876<br>-0.0486<br>-0.0941	0.5443<br>0.1326<br>0.1027<br>0.1018	(2.250, 4.389)<br>(-0.067, 0.456)<br>(-0.249, 0.156)<br>(-0.289, 0.107)	8.11e-07<br>0.167<br>0.640<br>0.363
tCO_2 (mmol/L)	(Intercept)<br>Baseline<br>Treatment<br>Period	10.9394<br>0.5352<br>0.6599<br>0.9401	4.2552<br>0.1739<br>0.8064<br>0.8064	(2.647, 19.269)<br>(0.196, 0.871)<br>(-0.915, 2.256)<br>(-0.628, 2.536)	0.01974<br>0.00694<br>0.42405<br>0.25923
CL- (mmol/L)	(Intercept)<br>Baseline<br>Treatment<br>Period	81.1406<br>0.2336<br>-0.0006<br>-2.1994	20.7760<br>0.1971<br>0.7649<br>0.7648	(40.845, 121.354)<br>(-0.148, 0.613)<br>(-1.569, 1.518)<br>(-3.716, -0.746)	0.00118<br>0.25284<br>0.99935<br>0.01064
GLU (mg/dL)	(Intercept)<br>Baseline<br>Treatment<br>Period	34.1094<br>0.6123<br>-2.4611<br>2.7611	13.5428<br>0.1507<br>1.6469<br>1.6469	(8.205, 61.571)<br>(0.309, 0.908)<br>(-5.670, 0.710)<br>(-0.474, 6.093)	0.021930<br>0.000807<br>0.153407<br>0.111924
CA (mg/dL)	(Intercept)<br>Baseline<br>Treatment<br>Period	4.3944<br>0.5277<br>-0.0072<br>0.0372	0.9179<br>0.0967<br>0.0778<br>0.0778	(2.574e+00, 6.171e+00)<br>(3.391e-01, 7.186e-01)<br>(-1.539e-01, 1.427e-01)<br>(-1.134388e-01, 1.912e-01)	3.23e-05<br>4.41e-06<br>0.927<br>0.635

BUN (mg/dL)	(Intercept)<br>Baseline<br>Treatment<br>Period	4.9611<br>0.5421<br>0.8111<br>1.5889	1.3588<br>0.0975<br>0.5226<br>0.5226	(2.227, 7.539)<br>(0.355, 0.739)<br>(-0.229, 1.822)<br>(0.546, 2.609)	0.00164<br>3.45e-05<br>0.13903<br>0.00739
CRE (mg/dL)	(Intercept)<br>Baseline<br>Treatment<br>Period	0.4713<br>0.4937<br>0.0450<br>-0.0550	0.1950<br>0.2057<br>0.0373<br>0.0373	(0.095, 0.845)<br>(0.090, 0.892)<br>(-0.030, 0.118)<br>(-0.1281, 0.018)	0.0268<br>0.0281<br>0.2446<br>0.1590
ALP (U/L)	(Intercept)<br>Baseline<br>Treatment<br>Period	1.5483<br>0.8982<br>2.0111<br>4.7889	5.0477<br>0.0878<br>0.9954<br>0.9954	(-8.225, 11.315)<br>(0.728, 1.067)<br>(0.063, 4.085)<br>(2.7612, 6.768)	0.762636<br>1.11e-08<br>0.059387<br>0.000163
ALT (U/L)	(Intercept)<br>Baseline<br>Treatment<br>Period	7.7869<br>0.7432<br>-0.5222<br>-2.0778	5.8564<br>0.2216<br>1.4769<br>1.4769	(-4.060, 19.268)<br>(0.313, 1.177)<br>(-3.562, 2.336)<br>(-4.908, 0.734)	0.20019<br>0.00376<br>0.72800<br>0.17750
AST (U/L)	(Intercept)<br>Baseline<br>Treatment<br>Period	10.3710<br>0.6565<br>-0.6333<br>-0.9667	5.1934<br>0.1410<br>2.0170<br>2.0170	(0.120, 20.795)<br>(0.370, 0.936)<br>(-4.578, 3.306)<br>(-4.932, 3.012)	0.059904<br>0.000227<br>0.757342<br>0.637859
TBIL (mg/dL)	(Intercept)<br>Baseline<br>Treatment<br>Period	0.4298<br>0.5492<br>-0.0478<br>0.0078	0.1752<br>0.1513<br>0.0753<br>0.0753	(0.096, 0.777)<br>(0.246, 0.847)<br>(-0.197, 0.100)<br>(-0.140, 0.154)	0.02333<br>0.00207<br>0.53419<br>0.91894
ALB (g/dL)	(Intercept)<br>Baseline<br>Treatment<br>Period	1.8175<br>0.5371<br>-0.0072<br>-0.0628	0.4755<br>0.1175<br>0.0518<br>0.0518	(0.918, 2.762)<br>(0.305, 0.761)<br>(-0.108, 0.095)<br>(-0.164, 0.042)	0.001339<br>0.000272<br>0.890686<br>0.241838
TP (g/dL)	(Intercept)<br>Baseline<br>Treatment<br>Period	2.4711<br>0.6379<br>-0.0467<br>0.0867	0.5971<br>0.0791<br>0.0461<br>0.0461	(1.258, 3.613)<br>(0.488, 0.801)<br>(-0.136, 0.047)<br>(-0.007, 0.181)	0.000679<br>3.25e-07<br>0.325404<br>0.077255

Table S4 - Mixed Linear Effects Model Results of SCFAs, sIgA, and Fecal Triglycerides

Test	Coefficient	$\hat{\beta}$	SE ($\hat{\beta}$)	95% CI	p-value
Acetate (uM/g)	(Intercept)	5.9377	1.4992	(3.070, 8.891)	0.000542
	Baseline	0.2381	0.0812	(0.077, 0.398)	0.008905
	Treatment	-2.3055	0.8812	(-3.981, -0.574)	0.017486
	Period	-0.2818	0.8812	(-2.082, 1.425)	0.752799
Propionate (uM/g)	(Intercept)	1.7334	0.3266	(1.076, 2.388)	1.60e-05
	Baseline	0.3083	0.0607	(0.188, 0.428)	7.89e-05
	Treatment	-0.5819	0.1946	(-0.968, 0.195)	0.00786
	Period	0.0614	0.1946	(-0.324, 0.447)	0.75612
Butyrate (uM/g)	(Intercept)	1.1510	0.4763	(0.228, 2.101)	0.02474
	Baseline	0.3065	0.0903	(0.127, 0.483)	0.00324
	Treatment	-0.2044	0.1949	(-0.589, 0.179)	0.30819
	Period	0.1916	0.1949	(-0.194, 0.564)	0.33858
sIgA (ug/ml)	(Intercept)	3.2385	1.9852	(-0.713, 6.978)	0.53323
	Baseline	0.5347	0.2463	(0.069, 1.027)	0.00174
	Treatment	0.4268	0.3058	(-0.173, 1.015)	0.90760
	Period	-0.1785	0.3058	(-0.776, 0.425)	0.30933
Fecal Triglycerides (mg/100mg)	(Intercept)	0.2288	0.3655	(-0.466, 0.926)	0.53545
	Baseline	0.7098	0.1975	(0.3223, 1.091)	0.00102
	Treatment	0.1650	0.1679	(-0.158, 0.498)	0.33281
	Period	0.2474	0.1679	(-0.088, 0.580)	0.14986

Table S5 - Mixed Linear Effects Model Results of Cytokines

Test	Coefficient	$\hat{\beta}$	SE ($\hat{\beta}$)	95% CI	p-value
GMCSF (pg/ml)	(Intercept)	8.24584	15.05226	(-21.246, 36.732)	0.596
	Baseline	0.92624	0.08601	(0.763, 1.098)	4.87e-06
	Treatment	11.52542	6.81733	(-1.707, 24.977)	0.129
	Period	-2.74792	6.81733	(-16.401, 10.339)	0.697

IFn-gamma (pg/ml)	(Intercept)	2.9737	2.6839	(-2.243, 8.319)	0.289676
	Baseline	0.7964	0.1471	(0.499,1.089)	0.000635
	Treatment	0.6629	1.5837	(-2.487, 3.854)	0.686537
	Period	-2.7654	1.5837	(-5.964, 0.218)	0.118931
IL-10 (pg/ml)	(Intercept)	7.7485	3.9303	(0.129, 15.469)	0.0662
	Baseline	0.6842	0.0849	(0.524, 0.847)	5.06e-07
	Treatment	1.6479	3.5283	(-5.510, 8.749)	0.6468
	Period	-4.4304	3.5283	(-11.415, 2.498)	0.2273
IL-12/p70 (pg/ml)	(Intercept)	1.8288	0.8353	(0.163, 3.480)	0.05463
	Baseline	0.6266	0.1277	(0.374, 0.885)	0.00118
	Treatment	0.3775	0.3213	(-0.256, 1.024)	0.27387
	Period	0.4425	0.3213	(-1.084, 0.177)	0.20580
IL-13 (pg/ml)	(Intercept)	2.56808	2.8248	(-2.947, 8.282)	0.378
	Baseline	0.86415	0.0387	(0.786, 0.942)	1.7e-08
	Treatment	-1.01333	2.1286	(-5.160, 3.146)	0.647
	Period	1.33833	2.1286	(-2.773, 5.444)	0.547
IL-13 (log-scale) (pg/ml)	(Intercept)	-0.0565	0.1806	(-0.407, 0.297)	0.762
	Baseline	1.0030	0.0605	(0.887, 1.124)	1.78e-07
	Treatment	0.0716	0.0527	(-0.033, 0.173)	0.212
	Period	-0.0164	0.0527	(-0.121, 0.085)	0.764
IL-1b (pg/ml)	(Intercept)	0.0578	0.4843	(0.932, 1.063)	0.907815
	Baseline	0.9447	0.1596	(0.616, 1.278)	0.000354
	Treatment	0.0346	0.1060	(-0.169, 0.244)	0.752550
	Period	-0.1371	0.10560	(-0.344, 0.079)	0.231930

IL-2 (pg/ml)	(Intercept)	0.5934	0.5400	(-0.446, 1.670)	0.30176
	Baseline	0.7999	0.1600	(0.483, 1.109)	0.00105
	Treatment	-0.0433	0.1287	(-0.300, 0.199)	0.74495
	Period	-0.1667	0.1287	(-0.419, 0.080)	0.23135
IL-4 (pg/ml)	(Intercept)	26.3030	11.6297	(3.110, 49.315)	0.046336
	Baseline	0.6466	0.0945	(0.464, 0.832)	0.000132
	Treatment	6.2325 -	5.4463	(-4.608, 16.943)	0.285560
	Period	1.8825	5.4463	(-12.390, 8.748)	0.738522
IL-5 (pg/ml)	(Intercept)	0.1241	0.2817	(-0.419, 0.668)	0.666
	Baseline	0.9176	0.0143	(0.890, 0.945)	3.93e-12
	Treatment	0.0454	0.2865	(-0.514, 0.613)	0.878
	Period	-0.1679	0.2865	(-0.731, 0.390)	0.574
IL-6 (pg/ml)	(Intercept)	0.6907	0.6616	(-0.621, 1.950)	0.317
	Baseline	0.8688	0.1042	(0.654, 1.077)	3.23e-05
	Treatment	0.3492 -	0.3995	(-0.435, 1.142)	0.408
	Period	0.4692	0.3995	(-1.238, 0.301)	0.274
IL-7 (pg/ml)	(Intercept)	7.4177	4.6276	(-1.578, 16.393)	0.1463
	Baseline	0.7015	0.2443	(0.231, 1.169)	0.0208
	Treatment	-1.0375	0.8050	(-2.630, 0.506)	0.2335
	Period	-0.3825	0.8050	(-2.010, 1.180)	0.6474
IL-8 (pg/ml)	(Intercept)	0.5473	0.6856	(-0.817, 1.874)	0.444
	Baseline	0.9179	0.0963	(0.734, 1.108)	1.21e-05
	Treatment	0.0204	0.2575	(-0.468, 0.520)	0.939
	Period	0.0571	0.2575	(-0.438, 0.564)	0.830
TNFa (pg/ml)	(Intercept)	2.8670	2.5269	(-2.195, 7.811)	0.28915
	Baseline	0.7210	0.3131	(0.107, 1.337)	0.05027
	Treatment	-0.5250	0.2033	(-0.925, -0.118)	0.03247
	Period	-0.7750	0.2033	(-1.177, -0.384)	0.00514

Lithocholic Acid (pg/ml)	(Intercept)<br>Baseline<br>Treatment<br>Period	1095.3046<br>0.3950<br>-87.8023<br>328.0238	364.1143<br>0.0858<br>327.6418<br>327.7033	(391.761, 1808.704)<br>(0.226, 0.566)<br>(-737.509, 567.368)<br>(-294.049, 967.144)	0.004788<br>0.000153<br>0.791714<br>0.329898
Nutriocholic Acid-Isomer (pg/ml)	(Intercept)<br>Baseline<br>Treatment<br>Period	6.2364<br>0.4584<br>-0.2717<br>-1.1873	2.7715<br>0.1516<br>2.5579<br>2.5649	(0.660, 11.548)<br>(0.165, 0.768)<br>(-5.342, 4.732)<br>(-6.313, 3.881)	0.03065<br>0.00593<br>0.91683<br>0.65010
7a_Hydroxy_3_oxo_5b_cholanoic Acid (pg/ml)	(Intercept)<br>Baseline<br>Treatment<br>Period	7.6346<br>0.2234<br>-1.4368<br>-0.6760	2.2449<br>0.1500<br>2.0167<br>2.0229	(3.099, 12.129)<br>(-0.083, 0.526)<br>(-5.419, 2.448)<br>(-4.587, 3.333)	0.00166<br>0.14685<br>0.48677<br>0.74268
Deoxycholic Acid (pg/ml)	(Intercept)<br>Baseline<br>Treatment<br>Period	67.9236<br>0.5740<br>-20.3103<br>33.4798	31.9391<br>0.1222<br>28.3843<br>28.4394	(2.870, 130.364)<br>(0.327, 0.820)<br>(-76.585, 37.266)<br>(-27.022, 88.685)	0.0404<br>3.75e-05<br>0.4789<br>0.2468
Ursodeoxycholic Acid (pg/ml)	(Intercept)<br>Baseline<br>Treatment<br>Period	2.1751<br>0.3288<br>1.0245<br>0.9950	1.76867<br>0.0548<br>1.9640<br>1.9640	(-1.435e+00, 5.607)<br>(2.231e-01, 0.433)<br>(-2.790e+00, 5.063)<br>(-2.814e+00, 4.832)	0.227<br>6.93e-07<br>0.605<br>0.616
Ursodeoxycholic Acid (log-scale) (pg/ml)	(Intercept)<br>Baseline<br>Treatment<br>Period	0.55596<br>0.51670.<br>0.04490<br>-0.0434	0.26699<br>0.124480<br>0.22404<br>0.22409	(0.012, 1.073)<br>(0.267, 0.764)<br>(-0.384, 0.474)<br>(-0.460, 0.398)	0.044676<br>0.000414<br>0.843912<br>0.849005
3a_6b_7bTrihydroxycholenoic Acid (pg/ml)	(Intercept)<br>Baseline<br>Treatment<br>Period	15.3271<br>0.3730<br>-6.9471<br>-11.199	7.2105<br>0.0396<br>8.1779<br>8.1779	(1.038e+00, 29.237)<br>(2.967e-01, 0.4533)<br>(-2.337e+01, 8.904)<br>(-2.727e+01, 4.863)	0.0405<br>2.99e-11<br>0.4012<br>0.1794
3a_6b_7b_Trihydroxycholenoic Acid (log-scale) (pg/ml)	(Intercept)<br>Baseline<br>Treatment	0.48929<br>0.64235<br>0.08911	0.34058<br>0.10696<br>0.30415	(-1.630e-01, 1.138e+00)<br>(4.3756e-01, 8.5109e-01)<br>(-5.1111e-01, 7.0968e-01)	0.159<br>6.83e-07<br>0.771

	Period	-0.0357	0.30420	(-6.2745e-01, 5.6053e-01)	0.907
Glycodeoxycholic Acid (pg/ml)	(Intercept)<br>Baseline<br>Treatment<br>Period	0.86449<br>0.11331<br>0.06012 -<br>0.22683	0.21678<br>0.06857<br>0.18308<br>0.18316	(0.44, 1.299)<br>(-0.029, 0.251)<br>(-0.288, 0.411)<br>(-0.584, 0.128)	0.000312<br>0.107652<br>0.746713<br>0.232665
Glycochenodeoxycholic Acid (pg/ml)	(Intercept)<br>Baseline<br>Treatment<br>Period	1.2459<br>0.3001<br>0.5371<br>0.0902	0.6643<br>0.0724<br>0.6412<br>0.6413	(-0.130, 2.501)<br>(0.151, 0.442)<br>(-0.694, 1.750)<br>(-1.145, 1.3643)	0.068954<br>0.000225<br>0.414674<br>0.889866
Glycochenodeoxycholic Acid (log-scale) (pg/ml)	(Intercept)<br>Baseline<br>Treatment<br>Period	-0.0645<br>0.63336<br>0.26215<br>-0.16417	0.2762<br>0.1502<br>0.2456<br>0.2461	(-0.612, 0.459)<br>(0.340, 0.935)<br>(-0.214, 0.751)<br>(-0.672, 0.327)	0.81653<br>0.00023<br>0.30198<br>0.51429
Glycocholic Acid (pg/ml)	(Intercept)<br>Baseline<br>Treatment<br>Period	2.4310<br>0.2944<br>0.1918<br>-0.7513	1.1282<br>0.1121<br>1.1329<br>1.1331	(2.091e-01, 4.720)<br>(7.779e-02, 0.514)<br>(-2.061e+00, 2.463)<br>(-3.020e+00, 1.550)	0.0379<br>0.0126<br>0.8665<br>0.5115
Taurodeozycholic Acid (pg/ml)	(Intercept)<br>Baseline<br>Treatment<br>Period	0.3403<br>0.2146<br>-0.0515<br>-0.0702	0.0801<br>0.0495<br>0.0573<br>0.0573	(0.186, 0.498)<br>(0.117, 0.309)<br>(-0.166, 0.056)<br>(-0.185, 0.043)	0.000195<br>0.000286<br>0.380782<br>0.236738
Taurodeozycholic Acid (log-scale) (pg/ml)	(Intercept)<br>Baseline<br>Treatment<br>Period	-0.3943<br>0.53746<br>-0.0564<br>-0.2901	0.1732<br>0.0990<br>0.1272<br>0.1279	(-7.383e-01, -0.056)<br>(3.364e-01, 0.737)<br>(-3.124e-01, 0.190)<br>(-5.372e-01, -0.036)	0.0295<br>1.25e-05<br>0.6630<br>0.0366
Taurocholic Acid (pg/ml)	(Intercept)<br>Baseline<br>Treatment<br>Period	0.6543<br>0.2793<br>-0.143<br>-0.213	0.2114<br>0.0464<br>0.1827<br>0.1827	(0.246, 1.056)<br>(0.186, 0.370)<br>(-0.484, 0.207)<br>(-0.573, 0.142)	0.00394<br>1.03e-05<br>0.44536<br>0.25843
Taurocholic Acid (log-scale) (pg/ml)	(Intercept)<br>Baseline<br>Treatment<br>Period	0.0634<br>0.7023<br>-0.2738<br>-0.2602	0.2803<br>0.1339<br>0.2752<br>0.2755	(-0.476, 0.610)<br>(0.434, 0.970)<br>(-0.824, 0.267)<br>(-0.804, 0.289)	0.823<br>3.84e-05<br>0.335<br>0.360

Table S6 – All Cytokines Outcome Data for 10 Randomly Selected Participants

Study Code (Participant)	Cohort	Treatment	Period	GMCSF pg/ml	IFn-Gamma pg/ml	IL-10 pg/ml	IL-12/p70 pg/ml	Il-13 pg/ml	IL-1b pg/ml	IL-2 pg/ml	IL-4 pg/ml	IL-5 pg/ml	IL-6 pg/ml	IL-7 pg/ml	IL-8 pg/ml	TNFa pg/ml
HOP-01	A	Control	1	171	17.96	24.23	6.59	9.34	2.61	3.55	65.81	3.76	6.12	20.86	3.25	11.48
		Cricket	2	189	15.51	15.38	6.05	10.93	2.36	2.55	87.49	3.26	5.35	17.39	4.03	9.87
		Baseline	0	145	14.51	12.26	4.75	7.8	2.55	2.25	66.03	2.54	4.68	12.26	2.64	8.05
HOP-02	A	Control	1	127	17.96	19.85	5.89	6.85	2.64	2.76	61.4	1.65	6.59	16.33	3.2	7.06
		Cricket	2	125	16.75	20.08	6.25	8.45	2.81	3.04	80.4	2.33	7.78	18.17	3.25	6.96
		Baseline	0	117	16.52	19.37	6.82	8.74	2.8	3.22	65.59	2.13	7.21	20.56	3.02	8.24
HOP-05	A	Control	1	217	11.04	24.64	5.41	15.04	2.93	2.31	103	0.72	0.34	20.21	8.54	9.28
		Cricket	2	244	7.79	24.94	5.39	14.27	2.99	2.56	89.2	1.26	0.26	18.23	8.64	6.86
		Baseline	0	225	10.55	18.02	5.7	11.63	3.27	2.9	128	1.13	0.34	16.83	7.42	9
HOP-08	A	Control	1	45.56	17.99	18.43	4.64	12.52	2.67	2.8	104	3.57	4.72	15.14	3.47	8.34
		Cricket	2	37.67	16.49	15.62	4.58	11.4	2.28	2.43	94.52	2.36	3.9	13.07	2.85	7.27
		Baseline	0	45.12	15.33	17.49	4.9	14.11	2.92	2.35	111	3.26	3.91	16.27	3.53	7.87
HOP-09	B	Control	2	53.22	5.87	7.17	3.49	3.33	1.84	1.49	79.32	1.41	2.17	16.71	2.97	6.72
		Cricket	1	65.92	9.73	8.65	5.06	3.45	2.29	1.71	94.3	1.43	3.68	14.97	4.12	7.08
		Baseline	0	53.14	9.56	9.65	4.17	3.86	1.47	1.72	81.48	1.67	2.96	15.69	3.84	6.68
HOP-11	B	Control	2	214	15.68	38.57	7.49	21.5	5.01	5.45	120	5.94	7.01	26.9	5.63	9.6
		Cricket	1	272	30.96	73.84	10.44	26.88	5.18	5.79	152	7.15	10.27	30.99	5.81	10.24
		Baseline	0	280	31.26	77.59	10.5	27.32	4.82	6.08	138	7.3	10.73	32.83	6.04	9.49
HOP-15	B	Control	2	172	17.19	35.44	5.98	161	3.09	3.84	131	31.15	7.14	24.79	14.58	7.3
		Cricket	1	193	20.11	40.7	6.23	143	3.69	4.14	120	32.41	7.59	22.31	13.03	7.6
		Baseline	0	184	16.15	36.15	5.26	177	3.22	3.59	124	34.56	5.77	22.14	15.49	6.94
	B	Control	2	61.01	3.31	8.87	3.14	3.76	1.47	1.32	42.05	0.28	1.8	9.72	3.53	5.9

HOP-16		Cricket	1	57.95	3.52	8.15	2.85	5.19	1.5	1.29	39.6	0.46	1.87	7.87	3.5	6.13
		Baseline	0	69.93	6.25	12.49	3.14	5.81	1.83	1.72	42.05	0.92	2.08	8.2	4.48	6.6
HOP-18	B	Control	2	199	9.36	14.8	3.45	8.5	1.59	3.25	89.84	1.74	3.38	16.63	2.56	7.14
		Cricket	1	198	7.47	11.34	3.4	9.72	1.81	3.17	106	0.8	2.71	13.99	3	7.02
		Baseline	0	176	10.55	13.76	3.71	8.35	2.01	2.7	112	1.26	3.41	15.56	2.55	6.68
HOP-19	B	Control	2	167.00	15.21	38.87	6.01	63.81	2.79	2.76	144.00	12.95	6.83	28.80	10.33	8.63
		Cricket	1	165	15.4	37.51	6.5	59.55	2.35	2.75	143	12.5	7.12	29.49	9.92	8.72
		Baseline	0	174	18.03	46.24	9.23	50.07	3.57	4.22	197	13.82	7.26	16.71	7.91	10.1

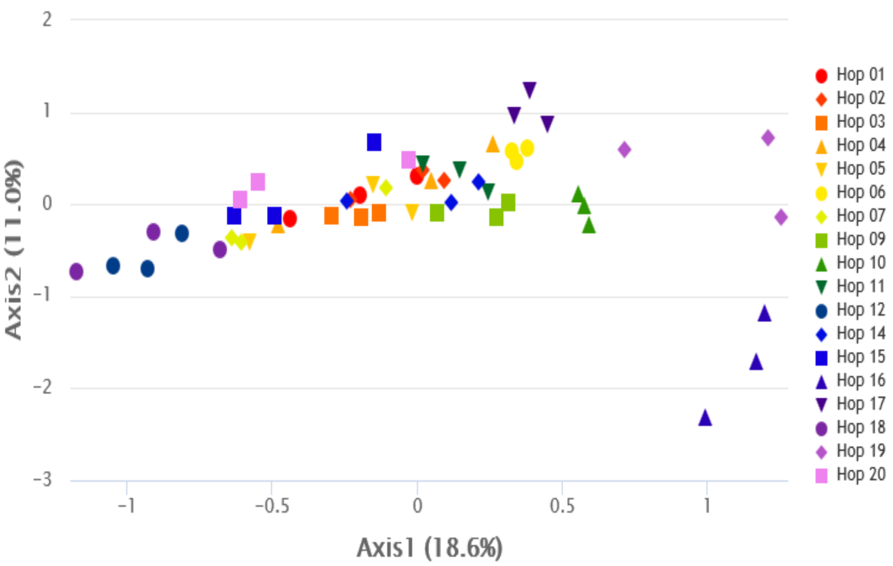

Figure S2 - Compositional Dissimilarity by Participant Across Treatment Periods

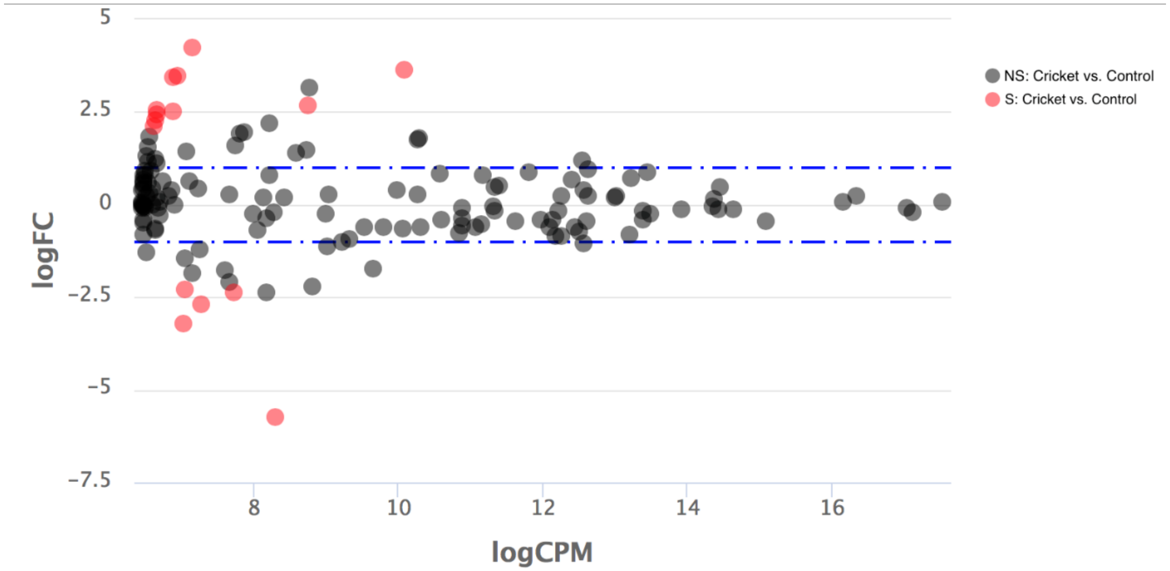

Figure S3 - Log-fold change in bacterial species abundance with cricket consumption compared to control diet (Genewise negative binomial GLM; q<0.1) [NS=not significant, S=significant]

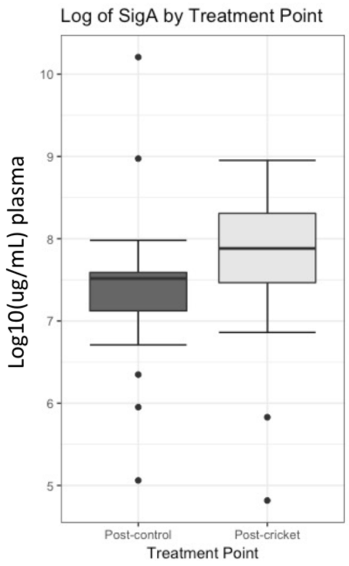

Figure S4 - Boxplots of Average sIgA Across Treatment Periods (μg/ml)
